# Early cost-utility analysis of hepatitis C virus testing for emergency department attendees in France

**DOI:** 10.1371/journal.pgph.0001559

**Published:** 2023-02-23

**Authors:** Nicolas Noiriel, Jack Williams

**Affiliations:** 1 London School of Hygiene & Tropical Medicine, London, England, United Kingdom; 2 Department of Health Service Research and Policy, London School of Hygiene & Tropical Medicine, London, England, United Kingdom; ICMR-National Institute for Research in Tuberculosis: National Institute of Research in Tuberculosis, INDIA

## Abstract

Testing for hepatitis C virus (HCV) is currently targeted towards those at high-risk in France. While universal screening was recently rejected, a growing body of research from other high-income countries suggests that HCV testing in emergency departments (ED) can be effective and cost-effective. In the absence of any studies on the effectiveness of HCV testing in ED attendees in France, this study aimed to perform an early economic evaluation of ED-based HCV testing. A Markov model was developed to simulate HCV testing in the ED versus no ED testing. The model captured costs from a French health service perspective, presented in 2020 euros, and outcomes, presented as quality-adjusted life years (QALYs), over a lifetime horizon. Incremental cost-effectiveness ratios (ICER) were calculated as costs per QALYs gained and compared to willingness-to-pay thresholds of €18,592 and €33,817 per QALY. Value of information analyses were also performed. ED testing for HCV was cost-effective at both thresholds when assuming ED prevalence of 1.1%, yielding an ICER of €3,800 per QALY. Testing remained cost-effective when the HCV prevalence amongst ED attendees remained higher than in the general population (0.3%). The maximum value of future research ranged from €10 to €79 million, depending on time horizons and willingness-to-pay thresholds. Our analysis suggests ED-based HCV testing may be cost-effective in France, although there is uncertainty due to the lack of empirical studies available. Further research is of high value, suggesting seroprevalence surveys and pilot studies in French ED settings are warranted.

## Introduction

### Context

The prevalence of hepatitis C virus (HCV) in France is low, with an estimated 133,500 people chronically infected in 2016 (0.3% of the general population aged 18 to 75 years) [1]. Still, it remains a public health issue in the country, with marginalized and stigmatized populations disproportionately affected [2–4], and an estimated 26,000 people remain unreached by current screening strategies [1].

Like in most high-income countries, HCV screening in France is targeted to people most at risk of infection [5]. However, the current HCV testing levels are insufficient to achieve World Health Organisation (WHO) targets to eliminate hepatitis C as a public health threat by 2030. These include a 90% and 65% reduction in respectively new cases of chronic HCV infections and HCV deaths by 2030, to which France has subscribed [6].

The limited success of current HCV testing strategies alongside the advent of low cost direct acting antiviral treatments (DAA) [6] provides scope for policy discussion and new research on alternative testing strategies. Despite evidence of the cost-effectiveness of universal screening in France [7, 8], the ‘Haute autorité de santé’ (HAS), the French scientific authority in charge of health technology assessment and public health recommendations, decided in 2019 to reject universal screening due to the lack of robust evidence and concerns around the feasibility of universal testing following HIV screening experiences [9]. Instead, they recommended maintaining a risk-based approach and intensifying screening in populations at increased risk, with further evaluations to identify prerequisites for better detection in those populations [10].

### Study rationale

A growing body of research from high-income countries suggests that testing people visiting emergency departments (ED) for HCV could help to identify and treat those with undiagnosed infections. Evidence shows that ED testing can be effective, feasible and acceptable to both patients and the healthcare workers [11–16]. Also, testing for HCV in ED patients was found to be cost-effective by recent model-based evaluations in the UK [17, 18], and in the US and Canada [19]. A large randomised controlled trial of ED testing has recently been announced in the US, with the aim of identifying the effectiveness of risk-based (targeted) testing versus universal (non-targeted) testing for HCV. This study will also include a cost-effectiveness analysis [20].

Whilst ED testing studies are available from other countries, there are no recent studies in France, meaning that the HCV prevalence in ED’s is uncertain. One French study from 1996 found that the HCV prevalence in the ED was higher than in the general population [21]. This relationship is common, with higher HCV prevalence found in ED’s in the US, Canada, UK, Ireland, and Germany [22].

This is likely because populations at high risk of infection (e.g. injecting drug users, migrants and homeless people) are more likely to attend ED’s, and may not engage with other services where HCV screening is performed. However, ED testing also captures those without risk factors who are unlikely to be tested otherwise. In US seroprevalence studies up to 31% people testing positive for HCV in ED’s were found to have no prior known or reported risk factor, hence the rationale for an ongoing US study to compare targeted versus non-targeted approaches to testing [20, 23–25].

In the absence of ED-specific HCV prevalence data, this analysis aimed to assess under which circumstances testing for HCV in ED settings might be cost-effective in France. The analysis also seeks to identify which parameters have the most influence upon the cost-effectiveness and to estimate the value of further research, by drawing on a value of information (VOI) framework. The VOI approach estimates maximum amount of money that might be invested to eliminate uncertainty in the cost-effectiveness decision, by assessing the probability of making the wrong decision (i.e. funding an intervention that is not cost-effective) and combining this with the opportunity cost associated with this decision (i.e. the value of the health that could have been gained if these resources had been allocated elsewhere). The results are expressed in monetary value as the expected value of perfect information (EVPI), and any study with costs exceeding the EVPI should not be considered a worthwhile investment.

## Methods

### Modelling approach

A decision analytical model was built to compare the current risk-based HCV testing approach with and without the addition of an opt-out HCV test for ED attendees for whom blood is taken for routine clinical care. The model run over a lifetime horizon (50 years), with a one-year cycle length. A health service perspective was taken, with costs reported in 2020 euros (€). Health outcomes are reported as quality-adjusted life years (QALYs), a measure of health outcomes that combines both the length of life and health related quality of life over time. This can be estimated weighting the time spent in the different health states by the health-related quality of life estimate of each health state. In the base case scenario, costs and outcomes were discounted at the same rate of 2.5% during the first thirty years, then at a rate progressively reduced to 1.5%, as per the HAS guidelines [26].

The model included adults aged 18 years or more presenting to the ED with no prior known diagnosis of HCV. They were assumed to be 48 years of age on average, based on data of ED attendees in France [27].

### Model structure

A decision tree was combined with a Markov model to simulate both the costs and outcomes associated with the testing strategy in terms of number of infected individuals detected and engaged in treatment. A Markov model was used to capture the long-term effects of diagnosis and treatment of chronic hepatitis C (CHC), with treatment reducing disease progression and subsequent mortality and morbidity in the cohort.

#### Decision tree

**Fig 1:**
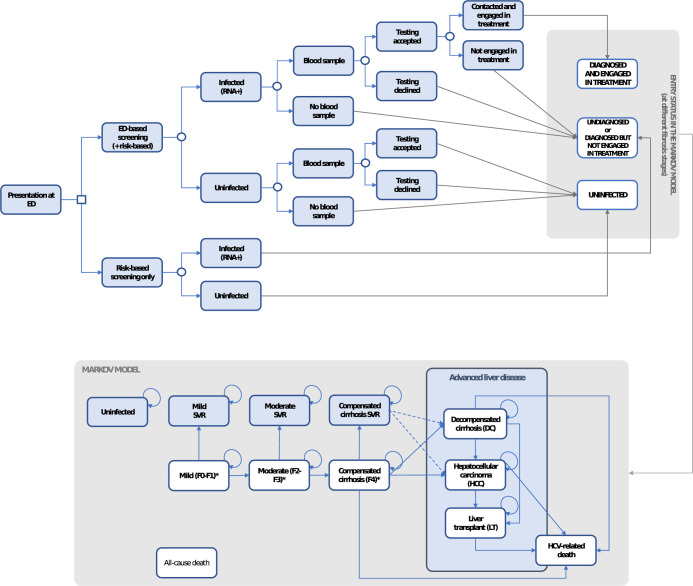
Model overview. *Individuals who are undiagnosed, untreated or who fail to achieve SVR after two treatment attempts. Dotted lines are indicative of slower progression.

The decision tree consisted of two arms, describing screening strategies of interest (**Fig 1**).

In the ED-based arm, individuals entered the decision tree as infected or uninfected, and were assumed to be offered testing if blood was taken as part of routine clinical care. Those who tested positive were offered treatment, with the possibility of achieving a sustained virological response (SVR), equivalent to cure. Those infected who did not receive a test entered the Markov model as undiagnosed. Those who tested positive but did not engage in treatment entered the transition model as diagnosed but with no treatment, and were assumed to face the same risk of disease progression as those undiagnosed. It was assumed that opportunity to be tested in ED’s occurred only once in a lifetime, but individuals still had the opportunity to test elsewhere as part of the risk-based background testing.

In the no ED testing arm, individuals entered the transition model either as uninfected, or as infected and undiagnosed, with an annual probability to be reached by background testing.

#### Markov model & health states

The Markov model was comprised of eight different health states, reflecting the natural history of HCV (**Fig 1**). It was adapted from models used previously in economic evaluations on HCV testing strategies [8, 17, 19]. Patients who remained untreated were at risk of progressing to more severe fibrosis stages and eventually compensated cirrhosis (CC). Patients with CC were then at risk of developing advanced liver disease (decompensated cirrhosis–DC, or hepatocellular carcinoma—HCC). Patients with DC or HCC might eventually require liver transplant.

Undiagnosed patients had an annual probability of receiving risk-based background testing, until they developed DC or more advanced liver damage, at which stage they were assumed to be aware of their status.

All individuals in the model had a risk of all-cause mortality [28]. From CC stage onwards, patients were assumed to face an additional risk of death due to their liver conditions.

### Parameters

#### ED prevalence

A targeted literature review was performed to identify data on the HCV prevalence in ED’s in high-income countries, and to what extent the HCV prevalence in ED’s exceeds that prevalence in the general population (**S1 Text**).

Our targeted review identified 19 ED testing studies, with two from Germany, one from Ireland, six from the UK and ten from the US. There were no studies identified from France.

The HCV prevalence was found to be higher in ED attendees than in the general population in all but one study [29]. A prevalence ratio was calculated (ED prevalence divided by estimated general population prevalence), with a weighted average of 4.6 across all studies. The prevalence ratio was estimated to be 3.8 across European studies, which was used in the analysis. This prevalence ratio was multiplied by with the general population HCV prevalence of 0.3% in France, to give an estimated ED prevalence of 1.1% [1]. The proportion of patients unaware of their status was assumed to be the same in ED attendees as in the general population (19.4%). Extensive sensitivity analyses were performed around these parameters given the uncertainty around them.

#### Further intervention effects

In the absence of data from France, the proportion of ED attendees from whom blood was taken for clinical routine care was assumed to be 40%, based on studies from other high income countries [17, 30]. The testing acceptance rate was estimated at 79%, based on HCV testing data from France [2] (**Table 1**).

**Table 1:** Model parameters.

Parameters	Mean value (SE)	Distribution	Reference
**Intervention effects**			
HCV RNA prevalence estimates			
Prevalence in the French general population	0.3% (0.1%)	Beta	[1]
Estimated prevalence ratio	3.8 (0.23)	Log-normal	Assumption based on targeted literature review
Prevalence in the ED attendee population	1.1%		Study calculations
Proportion of undiagnosed cases	19.4% (13.1%)	Beta	[1]
ED-specific prevalence estimate of undiagnosed HCV	0.23%		Study calculations
Proportion of routinely requiring blood sample	40.0% (20%-60%)^†^	Uniform	[17, 30]
Testing acceptance rate	79.0% (50%-95%)^†^	Uniform	[2]
Proportion of patients tested effectively engaged in care	33.0% (10.2%)	Beta	[17]
Fibrosis stage distribution at presentation			
F0-F1	40.5%	Dirichlet	[31]
F2	24.1%	Dirichlet	
F3-F4	35.3%	Dirichlet	
Annual probability of background testing	6.2% (3.8%)	Beta	[32]
**Transition probabilities per cycle**			
From F0 to F1	0.107 (0.005)	Beta	[33]
From F1 to F2	0.082 (0.004)	Beta	
From F2 to F3	0.117 (0.006)	Beta	
From F3 to F4	0.116 (0.007)	Beta	
From F4			
…to decompensated cirrhosis (DC)	0.05 (0.038)	Beta	[34]
…to hepatocellular carcinoma (HCC)	0.017 (0.013)	Beta	[35]
…to HCV-related death	0.01 (0.006)	Beta	[36]
From DC			
…to HCC	0.017 (0.013)	Beta	[35]
…to liver transplant (LT)	0.12 (0.092)	Beta	[37]
…to HCV-related death	0.13 (0.01)	Beta	[38]
From HCC			
…to LT	0.17 (0.13)	Beta	[39]
…to HCV-related death	0.43 (0.03)	Beta	[38]
From LT			
…to HCV-related death (first year)	0.158 (0.01)	Beta	[40]
…to HCV-related death (following years)	0.039 (0.001)	Beta	
From F4 SVR			
…to DC (RR, respective to non-SVR probability)	0.07 (0.043)	Log-normal	[41]
…to HCC (RR respective to non-SVR probability)	0.19 (0.092)	Log-normal	
Chance of achieving SVR			
After first treatment in F0-F3	0.928 (0.013)	Beta	[42]
After re-treatment in F0-F3	0.93 (0.01)	Beta	
After first treatment in F4	0.908 (0.026)	Beta	
After re-treatment in F4	0.855 (0.042)	Beta	
All-cause mortality			
45 to 49 years	0.002		[28]
50 to 54 years	0.003		
55 to 59 years	0.005		
60 to 64 years	0.008		
65 to 69 years	0.011		
70 to 79 years	0.018		
80 to 89 years	0.057		
90 to 110 years	0.183		
**Health state utility scores**			
F0-F1	0.82 (0.1^‡^)	Beta	[31]
F2	0.82 (0.1^‡^)	Beta	
F3—Compensated cirrhosis (F4)	0.76 (0.1^‡^)	Beta	
Decompensated cirrhosis	0.60 (0.1^‡^)	Beta	[43]
Hepatocellular carcinoma	0.60 (0.1^‡^)	Beta	
Liver transplant (first year)	0.55 (0.1^‡^)	Beta	
Liver transplant (following years)	0.82 (0.1^‡^)	Beta	
SVR in mild fibrosis (F0-F1)	0.95 (0.1^‡^)	Beta	
SVR in moderate fibrosis and cirrhosis (F2-F4)	0.85 (0.1^‡^)	Beta	
**Cost parameters (cost year)**			
**Screening costs (2020)**			
Anti-body test cost	13 (8.7)	Gamma	[44]
RNA-positivity test cost	52 (19.9)	Gamma	
Contacting costs	10 (6.4)	Gamma	Assumption
Outpatient evaluation (prior to treatment)	138 (78.3)	Gamma	Assumption
Background testing costs	25 (9.6)	Gamma	Assumption
**Treatment costs (2020)**			
Drug costs	24,836 (12,400–37,200) ^†^	Uniform	[45, 46]
Monitoring costs	138 (78.3)	Gamma	Assumption
**Health-state specific costs (2015)** ^§^			
F0-F2			
Ambulatory costs (treatment naïve)	72 (10)	Gamma	[8]
Ambulatory costs (after treatment failure)	54 (12)	Gamma	
Hospitalization costs	286 (1,117)	Gamma	
F3			
Ambulatory costs (treatment naïve)	131 (23)	Gamma	
Ambulatory costs (after treatment failure)	88 (15)	Gamma	
Hospitalization costs	286 (1,117)	Gamma	
F4			
Ambulatory costs (treatment naïve)	234 (21)	Gamma	
Ambulatory costs (after treatment failure)	73 (18)	Gamma	
Hospitalization costs	1,330 (3,834)	Gamma	
DC			
Ambulatory costs	99 (22)	Gamma	
Hospitalization costs	8,343 (9,427)	Gamma	
HCC			
Ambulatory costs	99 (22)	Gamma	
Hospitalization costs	12,065 (11,951)	Gamma	
Liver transplant (first year)			
Ambulatory costs	-	Gamma	
Hospitalization costs	57,546 (41,427)	Gamma	
Liver transplant (following years)			
Hospitalization costs	5,593 (11,426)	Gamma	

RNA = ribonucleic acid. LR = literature review. HCV = hepatitis C virus. RR = Relative risk. SVR = Sustained virological response. SE = standard error. †Range of values considered within a uniform distribution, instead of SE. ‡Due to lack of information about SE, sample size, beta or alpha, assumption of 0.1 was made to capture uncertainty. §Adjusted to 2020 costs, using the Hospital and community health services Pay and prices Index Inflation, from the French Office for national statistics (INSEE) (1.03).

It was assumed that 33% of patients tested in the ED would receive treatment, based on a UK study [17] and linkage to care values identified in the targeted literature review (ranging from 30% to 94%) [47–52]. For those detected through background testing, all were assumed to engage in care. Chance of being reached by background testing was estimated at a rate of 6.2% derived from a French study on laboratories’ HCV and HBV testing activity [32] (**Table 1**).

The distribution of fibrosis stages amongst those with HCV was derived from a previous French study [31] (**Table 1**). No stage beyond compensated cirrhosis (CC) was considered at presentation to ED, assuming that people with advanced liver disease would be aware of their status.

#### Treatment outcomes

For those receiving treatment, it was assumed 91% to 93% of patients would achieve SVR (depending on their fibrosis stage at treatment initiation), upon which they had no further disease progression, except for those in F4, who might progress to DC or HCC, but at a reduced rate. Those without SVR were retreated once, at a lower probability of SVR, depending on fibrosis stage (**Table 1**). The model did not consider the risk of reinfection or of onward HCV transmission.

#### Transition probabilities & mortality rates

The transition probabilities for disease progression were taken from the literature (**Table 1**). These have been used in other cost-effectiveness analyses of HCV testing in France [8], apart from updated estimates for fibrosis stages [33, 53] and risk of death following liver transplantation [40].

#### Utilities

Health utility scores were obtained from the two most recent studies evaluating health-related quality of life in patients with CHC in France (**Table 1**), one focusing on fibrosis stages [31], the other on advanced liver disease [43]. Those studies elicited health-state evaluation using a EuroQol-5D questionnaire, with a time trade-off (TTO) value set used for France [54], as per HAS recommendations [26].

#### Costs

Screening, treatment and health-state specific costs were included (**Table 1**). Screening costs included an HCV antibody test, and for those antibody positive, a subsequent RNA test. Test costs were taken from reimbursement data from the French national sickness fund [44]. It also included time to contact RNA-positive patients and to link them to care. This process was assumed to cost €10.00, under the assumption of an average of 26 minutes to contact each patient (whether contact was successful or not), derived from a previous cost-effectiveness analysis on HCV ED-based testing [17, 55] and of a nurse hourly cost of €24/hour. Patients successfully contacted and receiving treatment were assumed to all undergo an outpatient evaluation, which was estimated to equate to three consultation visits (evaluation, follow-up during treatment and after treatment), plus extra-fees due to complexity (€ 138.00 in total) [56]. For patients reached by background testing, cost was assumed to amount to a GP appointment (€ 25.00), in addition to test costs, if infected (**Table 1**). No additional cost for blood collection was considered, as blood samples were taken for routine care.

Drug prices were estimated using public database available from the French national sickness fund, with an average cost of €24,836 assumed for course of DAA treatment [57, 58] (**Table 1**).

Health-state specific costs covered outpatient care (ambulatory) and hospitalization costs associated to being in a HCV-state. Costs were derived from the 2018 French model-based CUA looking at HCV screening, except that increased costs associated to death occurring in hospital were not taken into account [8], because share of in-hospital deaths was unknown. Ambulatory cost estimates differed as to whether the patient was treatment naive or experienced SVR failure (**Table 1**).

As instructed by the HAS [26], all costs were adjusted, using the Hospital and community health services pay and prices index inflation [59].

### Model analyses

Incremental cost effectiveness ratios (ICER) were calculated as costs per quality-adjusted life years gained (€/QALY). In the absence of an explicit willingness-to-pay (WTP) threshold in France, two thresholds were used. A threshold of one-time GDP per capita (€33,817), the lower limit previously recommended by WHO [60], and a more conservative value of €18,592, based on the health opportunity cost estimated for France by Woods et al [61] and adjusted to 2020 prices [62]. This equates to 0.55 time GDP per capita.

#### Sensitivity analysis

Both deterministic and probabilistic sensitivity analyses were performed to assess the impact of parameter uncertainty. In the deterministic sensitivity analysis (DSA), the prevalence, DAA price, the cost of antibody test, the rate of background testing and the proportion of positive patients engaged in care were varied. Regarding rate of background testing, lower and upper bounds of 4% and 19% used in two French studies were considered, differentiating proportion of people reached by risk-based strategy, according to presence of risk factors or not [63, 64]. The proportion of patients engaged in care was varied from 20% to 60% based on the value range retrieved in the targeted literature review. In accordance with HAS guidelines [26], discount rate was also varied, using 0% and 4.5% for both costs and QALY.

In the absence of French studies of ED testing, a threshold analysis sought to provide minimum prevalence estimates under which the intervention remains cost-effective. A range of HCV prevalence was considered, and resulting ICERs were compared WTP thresholds, to account for potential regional variations in prevalence [65].

In the probabilistic sensitivity analysis (PSA), appropriate distributions were assigned to each parameter (**Table 1**). The analysis ran 10,000 Monte Carlo simulations, with each parameter randomly sampled and corresponding ICERs calculated.

#### Value of information analysis

Using the iterations from the PSA, the expected value of perfect information (EVPI) was estimated using the average net health loss that could occur if the wrong decision were made. The EVPI per person was multiplied by the estimated number of beneficiaries per year, for each year of the time horizon. Approximately 10,662,000 different patients aged between 18 and 80 years visit the ED each year in France [27, 66]. We considered the time horizon of the intervention over 2 years in the base case, but also considered values of 3, 5 and 10 years. These were deemed to be relevant for decision making, given 2030 elimination targets. A 2.5% discount rate was included, as per HAS guidelines [26].

## Results

### Base case results

ED-based screening was found to be more effective in detecting new cases, than current risk-based approach. For every 100,000 individuals presenting to ED’s, ED-based screening detected 70 additional cases, of whom 23 effectively engaged in care.

This reduced morbidity and mortality, and for every 100,000 ED attendees, 40 years less were lived with DC, HCC and LT, and 5 HCV-related deaths were averted.

ED testing was associated with an additional €2.01 for each person being tested, due to extra testing and treatment-related costs, outweighing savings in health-state related costs, and yielded a gain of 0.0005 QALYs per person. This gave an ICER of € 3,813 per QALY gained (**Table 2**), which meant the intervention was highly cost-effective at both WTP thresholds in the base case.

**Table 2:** Base-case cost-effectiveness results per person attending the ED.

Screening strategies	Mean cost (€)	Mean QALYs	ICER (€/QALY)
No ED testing	133.84	22.3986	
ED testing	135.85	22.3992	
**Incremental**	**2.01**	**0.0005**	**3,813**

ICER = incremental cost-effectiveness ratio.

**Fig 2:**
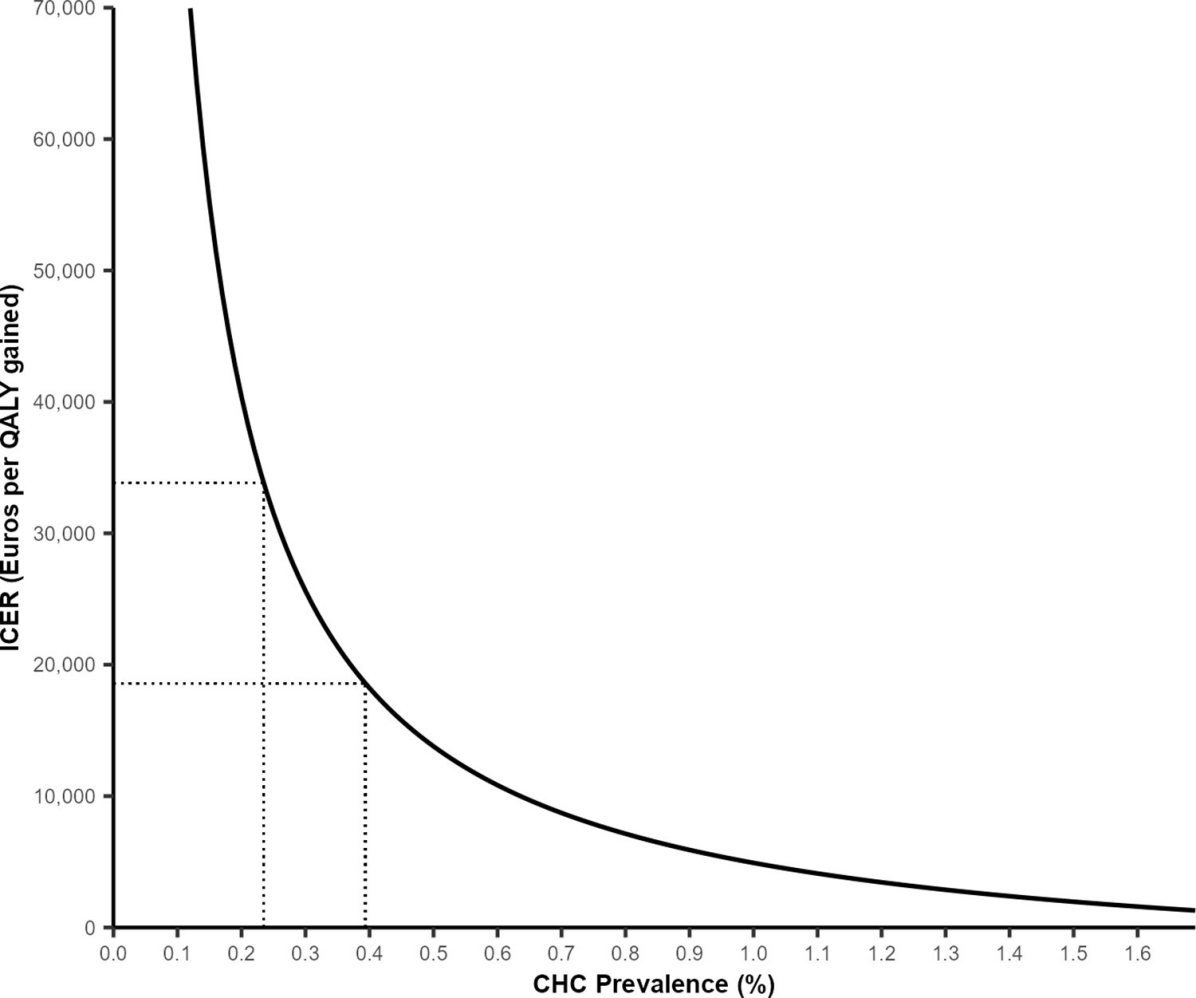
Threshold analysis. Dotted lines indicate WTP thresholds of € 18,592 and € 33,817.

A threshold analysis identifying the lowest ED prevalence at which testing would remain cost-effective found that testing was cost-effective when the prevalence exceeded 0.39%, for both WTP thresholds (**Fig 2**). When considering the higher WTP threshold of € 33,817, testing remained cost-effective when the prevalence was 0.24% or higher.

### Sensitivity analysis

#### Deterministic sensitivity analysis

**Fig 3:**
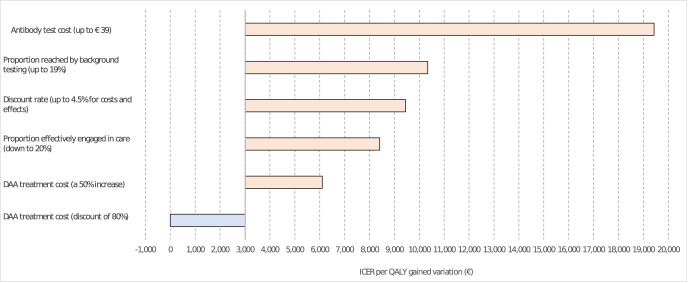
Tornado—One-way analysis.

The one-way analyses found that the ICER was most sensitive to an increased cost of antibody test. When the cost was at least the triple of initial assumption, the ICER ended higher than the lowest WTP threshold (€19,427). A lower proportion of patients reached by background testing, higher discount rates of 4.5%, a higher DAA cost and a lower proportion of patients engaged in care, all increased the ICER to between €8,408 to €10,336 (**Fig 3**). None of these altered our final conclusions about cost-effectiveness, as none increased the ICER above either of the WTP thresholds. A discount of 80% in DAA prices reduced the ICER to just €138.

When varying simultaneously the antibody test cost and, alternatively, the probability of being reached by background testing or the probability of engaging in care, a two-way analysis found that the ICER remained always below the one-time GDP threshold (€33,817), and below the lowest WTP threshold (€18,592) in most combinations. Only some combinations where both parameters took simultaneously extreme values of at least twice those of base case settings increased the ICER above €18,592 (**S1 Fig**).

#### Probabilistic sensitivity analysis

**Fig 4:**
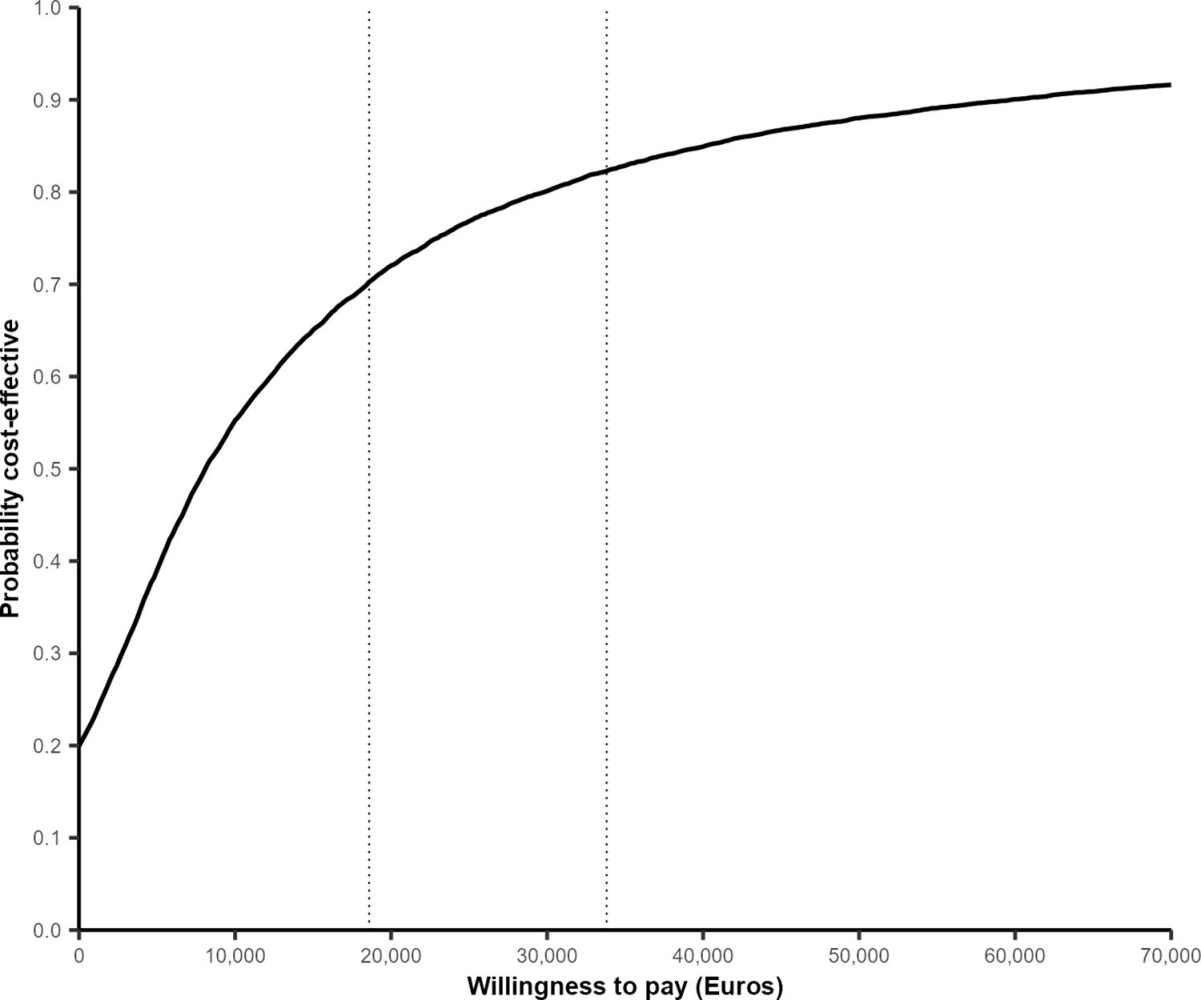
Cost-effectiveness acceptability curves (CEAC). Dotted lines indicate WTP thresholds of € 18,592 and € 33,817.

**Fig 5:**
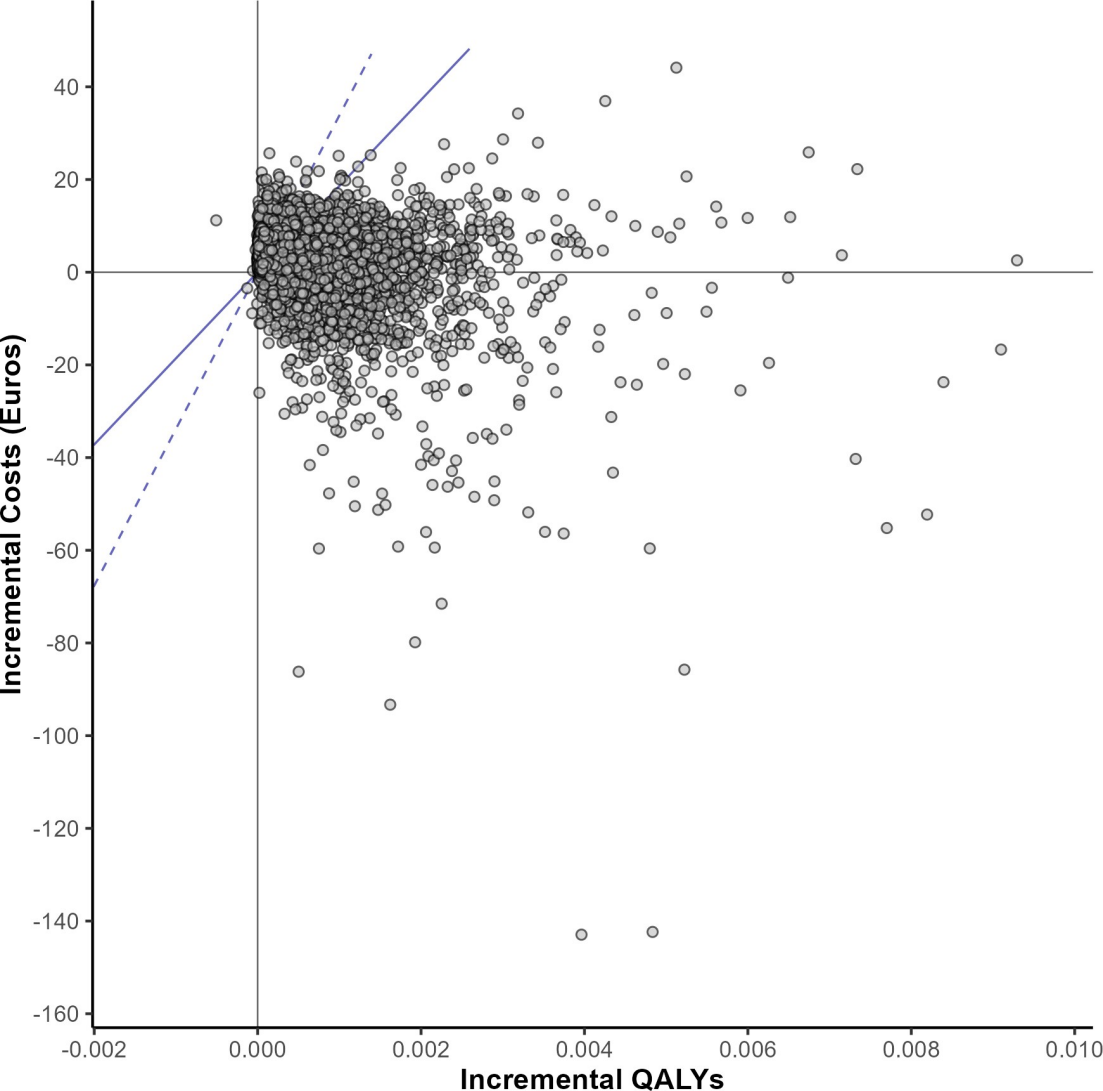
Cost-effectiveness plane. Lines indicate WTP thresholds of € 18,592 (plain) and € 33,817 (dotted).

The intervention was likely to be cost-effective in the base case analysis. At the WTP thresholds of €18,592 and €33,817, testing was cost-effective in 70% and 82% of simulations (**Fig 4**). The mean probabilistic ICER was €4,070, which is slightly higher than the deterministic ICER, due to some uniform distributions for parameters not being centred around the deterministic mean. Regardless of WTP threshold, the intervention had a 20% probability of being the dominant strategy (more effective, less costly), while there was very little chance of being dominated (less effective, more costly) (**Fig 5**).

#### Expected value of perfect information

The population level EVPI was €17 and €10 million when considering all ED attendees across France for a period of 2 years, for WTP of respectively € 18,592 and € 33,817. This corresponds to a value of information per person attending the ED of €0.83 and €0.49, respectively (**S2 Fig**). When considering longer time horizons, the population EVPI increased to €26-€15 million (3 years), €42-€25 million (5 years) and €79-€47 million (10 years), at the higher and lower WTP thresholds respectively (**S1 Table**).

## Discussion

### Main findings

Our findings demonstrate that HCV testing in the ED could be cost-effective in France, although there is a high degree of uncertainty given the absence of any empirical testing data from the ED. Using epidemiological data from France, alongside evidence of the effectiveness of ED testing from other high-income countries, our results suggest that the ED could be a well-positioned setting to reach those infected with HCV, at an acceptable cost for the French healthcare system. Testing remained cost-effective as long as the prevalence in ED attendees remains higher than in the general population. These results might be insightful from both a national perspective, as well as from a local one, as the HCV prevalence might differ markedly across regions and locations in France.

A recent economic evaluation reported that universal HCV testing may be cost-effective in France, however this testing approach has been rejected by decision makers due to a lack of empirical evidence, and because of uncertainties of the effectiveness of universal testing based on HIV testing experiences. Testing in the ED could be one way in which HCV testing is increased to include those who are currently unlikely to receive testing, without the need for a formal universal testing strategy. There is currently a lack of empirical evidence of ED testing in France, but our findings suggest that such a study would be valuable. We show there is a high value placed upon further research in this area, in order to reduce the uncertainty around the HCV testing decision, and help ensure the most appropriate testing policy is pursued. The high value of research identified in our EVPI analysis is due to the high number of people who could benefit from an ED testing intervention, but also due to the considerable uncertainty around whether testing is likely to be cost-effective in France.

Our study also adds to previous economic evaluations of HCV testing in EDs by incorporating cost, utility and testing parameters specific to France. These parameters are likely to differ across countries, particularly as costs derived from the US and Canada are likely to be higher. We have also identified the key factors when considering ED-based HCV testing in France, with the infection prevalence amongst attendees and the cost of HCV antibody tests particularly important. If lower antibody test prices and/or lower DAA prices can be obtained, then the affordability as well as cost-effectiveness of the intervention would be significantly improved. The introduction of HCV testing in the ED would represent a significant increase in testing, which may give the possibility for lower test prices (per unit) to be negotiated with increased testing volumes.

### Comparisons with other research

Our findings are consistent with previous economic evaluations on opt-out ED-based screening schemes, considered in the UK, the US and the Canadian settings [17–19]. In the UK, ED testing was highly cost-effective, with HCV testing costing £8,019/QALY, under the assumption of 1.4% CHC prevalence in ED’s [17]. A follow up cost-effectiveness analysis from two UK cities found similar ICERs of £7,177 and £12,387 for testing in Leeds and London respectively [18]. Testing remained cost-effective at a minimum prevalence of 0.5% in both cities, slightly above the UK general population prevalence (0.3%) [67]. In Canada and the US, one study found that ED testing was cost-effective, in both countries, with a reference ED prevalence value of 1.8%: at CAN$25,584/QALY and US$42,615/QALY in the birth-cohort option, and at CAN$19,733/QALY and US$32,187/QALY in the general population one. Again, the intervention remained cost-effective in Canada, at a minimum prevalence of 1%, slightly above the Canadian general population prevalence (0.8%).

The similarities in findings is unsurprising given that our evaluation used a similar model and in some instances, used parameters derived from other countries, where these were unavailable from France. The ED is known to be a touchpoint for marginalized and underserved communities, at increased risk of HCV infection and less likely to engage with conventional care models. Even though epidemiological context differs across countries, this pattern in use of ED services is likely to remain common across high-income countries.

### Limitations

This study has numerous limitations that might affect the interpretation of the findings.

EVPI estimate bears some limitations that might lead to an actual lower value. First, HCV prevalence in France is declining over time, which was not incorporated into the VOI analysis. Second, there is uncertainty in the estimate of annual number of ED attendees. Some individuals might attend ED services several times in a year. Ultimately, the relevance of the VOI analysis is always conditional on how well the decision model was set and the extent to which parameter uncertainty was well incorporated by assigning appropriate distributions to each parameters. Limitations of present study in that regard are inherent to secondary data research, where parameter estimates were taken from the literature.

Our study sought to draw on best existing evidence from France. Still, uncertainty around the parameter values remains. Sensitivity analyses aimed to account for this, however there remain several assumptions made in the absence of data. There is particular uncertainty around the prevalence of HCV amongst ED attendees in France, and also the intervention effectiveness following diagnosis (e.g. the proportion of patients engaged in care after being detected). The ED-specific prevalence estimate is a key parameter in the model and to which results are sensitive. Findings from other studies have shown an elevated HCV prevalence in ED attendees but these have mostly been performed in urban areas. The robustness of our results could be strongly improved by a more accurate measure taken from seroprevalence surveys conducted specifically in France.

This model is also simpler compared to other economic evaluations of HCV testing. First, it does not incorporate the impact of risks factors, such as drug or alcohol intake, or the existence of HIV/HBV co-infection which may alter the effectiveness of screening or disease progression, and this heterogeneity amongst risk groups could impact the cost-effectiveness estimates. Second, the model does not account for HCV transmission, either as reinfection for those achieving SVR, or the benefit of reduced onward transmission. Third, the model does not allow for people to be detected beyond the stage of CC. Fourth, our study takes a health system perspective, excluding indirect and future unrelated costs and benefits, albeit these would likely make such an intervention even more cost-effective. Finally, the model only evaluated HCV testing, while additional blood-borne virus testing for HIV and HBV may be of interest too, with an integrated testing approach recommended by the European Centre for Disease Control [68]. The value of research into a blood borne virus testing strategy would be even higher than that for HCV alone, although considering testing for multiple infections will add complexity to the decision making process, particularly as some areas may have a higher prevalence of some blood-borne viruses compared to others [69].

### Implications for policy and future research

Our results can be useful to illuminate the current debate around HCV screening policy in France. While HAS recently dismissed universal screening in favour of intensification of current risk-based testing approach, opt-out ED-based screening could be considered a middle-ground, by providing testing in settings that are open to everyone but in which those at higher risk of HCV are more likely to attend.

Unlike the HAS objections against universal screening, it can be argued that other high-income countries have set a precedent for ED screening, where early evidence has shown it to be feasible, acceptable, and a cost-effective way to detect undiagnosed HCV. Whilst it remains too early to recommend any ED testing policies due to a lack of empirical evidence from France, our study indicates that the value of further research is likely to be high.

Future studies should address several key uncertainties. Firstly, studies should estimate the HCV prevalence in French ED settings, and ideally seroprevalence surveys should be undertaken in multiple sites across areas in France. Future pilot studies should also seek to confirm the feasibility and acceptability of HCV screening in the ED, as well as collecting data on the effectiveness of the intervention to link those who are diagnosed into care and onto treatment, as this is an area of uncertainty which is important to the model results. Barriers to effective linkage to care might be substantial in ED settings, owing to socio-economic profiles of individuals more likely to have no health coverage, and the difficulties in linking patients following a one-off ED visit.

## Conclusions

Our early findings suggest that ED-based HCV screening could be cost-effective in France, using epidemiological data from France and assumptions around the effectiveness of ED-testing from other high-income countries. There remains considerable uncertainty around whether such an intervention would be cost-effective in France, however, our findings show that future research would be of a high value, and therefore this might be a policy option worth investigating further. This is particularly true given HAS reservations about providing universal screening for HCV in France.

If future studies are performed, then these should seek to address uncertainties around the prevalence of HCV amongst ED attendees in France, and consider the acceptability and effectiveness of an ED-based screening strategy in terms of linking patients onto treatment. There may also be potential to consider a blood borne virus screen in ED’s, which would also include HIV and HBV.

## Supporting information

S1 Text Targeted literature review. (DOCX)

S1 Fig Two-way sensitivity analysis. (DOCX)

S2 Fig Per-patient EVPI according to varying willingness-to-pay thresholds. (TIFF)

S1 Table Population EVPI across varying willingness to pay thresholds. (DOCX)
