# Supplementary material for: Early cost-utility analysis of hepatitis C virus testing for emergency department attendees in France
Source: PLOS Glob Public Health. 2023 Feb 23;3(2):e0001559. doi: 10.1371/journal.pgph.0001559 (PMC10021824; doi:10.1371/journal.pgph.0001559)
Supplement: S1 Text — (DOCX) [file pgph.0001559.s001.docx]

### S1 Text Targeted literature review

#### Search strategy

| **Population** | Patients attending ED settings |
| --- | --- |
| **Intervention** | - |
| **Comparator** | General population |
| **Outcome** | Prevalence, burden of disease of HCV (acute, chronic) |
| **Study design** | Observational studies (cohort studies, cross-sectional studies), trials, systematic reviews/meta-analyses, feasibility studies, pilot studies |

We searched the databases EMBase, MedLine, Cochrane, Web of science, Scopus, and Open Grey, using the terms:

| **Concepts** | Hepatitis C | Prevalence | Emergency department | High-income settings |
| --- | --- | --- | --- | --- |
| **Terms** | Hepatitis C  HCV  CHC | Prevalence  Prevalent  Seroprevalence  Frequency  Frequent  Burden of disease  Morbidity | Emergency departments, settings, units, wards, services; ED  Accident & emergency departments, settings, units, wards, services, A&E  Urgent units  Trauma centers | High-income countries  OECD  EU/EEA/European union  United States  United Kingdom  France |

We also looked for additional resources from institutions known to be involved in hepatitis research and policy in France:

- ‘Haute autorité de santé’ (HAS), the French scientific authority in charge of health technology assessment and public health recommendations,
- ‘Agence nationale de recherche sur le sida’ (ANRS), a public research agency specialized in blood-borne viruses in France,
- ‘Assocation française pour l’étude foie’ (AFEF), the French national medical society for hepatology.

#### Identification & selection of studies

Inclusion criteria:

- Measure of prevalence of HCV infections (acute, chronic)
- Measure specific to ED settings
- Measure of prevalence regardless of risk-based assessment (universal)
- In high resource settings, in the sense of the World Bank

Exclusion criteria:

- Measure of prevalence of other hepatitis than C (A-E)
- Prevalence of HCV limited to co-infections (HIV, HBV)
- Absence of prevalence specific to ED settings
- Prevalence limited to some subpopulations (pregnant women, migrants, drug users) or to specific risk factors, or birth cohorts.
- In low or middle resource settings, in the sense of the World Bank

Searches were run in June 2021. Research was limited to the 2010-2021 period. Data taken from studies published in previous years were deemed too old to reflect the changing epidemiologic pattern occurring in high income countries and the impact of the introduction of DAA treatments on a large scale.

Research was also restricted to papers published either in English or in French.

#### Main results

19 studies were identified from a range of high-income countries: two from Germany, one from Ireland, six from the UK and ten from the US. There were no studies identified from France.

Synthesis of results of non-systematic targeted literature review

| **Country** | **Number of studies** | **Range of sampling period** | **Anti-body prevalence in the gen. population (country reference)** | **Range of anti-body prevalence estimates in ED (from studies)** | **Range of RNA prevalence estimates in ED (from studies)** |
| --- | --- | --- | --- | --- | --- |
| Germany | 2 | 2008-2010 | 0.5% (0.4-0.6) | 2.6%-3.4% | 1.6% |
| Ireland | 1 | 2014-2015 | 0.8% (0.5-1.2) | 5.1% | - |
| United Kingdom | 6 | 2014-2016 | 1.2% (0.9-1.8) | 1.2%-2.4% | 0.5%-1.8% |
| United Sates | 10 | 2008-2020 | 1.7% (1.4-2.0) | 1.6%-14% | 0.2%-11.1% |

#### Detailed results

| **Study title** | **Authors; year of publication** | **Sampling period** | **Country** | **Screening approach** | **Sample size** | **Prevalence ratio*** | **Anti-body prevalence in the gen. pop.** | **Anti-body prevalence in ED** | **RNA prevalence in ED** |
| --- | --- | --- | --- | --- | --- | --- | --- | --- | --- |
| Unexpected high prevalence of hepatitis C in a densely populated metropolitan area of Germany | Dogiami, et al; 2013 | 2009-2010 | Germany | Universal | 8,435 | 6.8 | 0.4%-0.63% | 3.4% | 1.62% |
| High prevalence of anti-HCV antibodies in two metropolitan emergency departments in Germany: a prospective screening analysis of 28,809 patients | Vermehren, et al; 2012 | 2008-2010 | Germany | Universal | 28,809 | 5.2 | 0.4%-0.63% | 2.6% (2.4-2.8) | 1.6% (1.5-1.8) |
| Opt-Out Panel Testing for HIV, Hepatitis B and Hepatitis C in an urban emergency department: a pilot study | O'Connell, et al; 2016 | 2014-2015 | Ireland | Universal | 8,839 | 6.0 | 0.5%-1.2% | 5.1% |  |
| HIV/HCV/HBV testing in the emergency department: a feasibility and seroprevalence study | Bradshaw, et al; 2018 | 2015-2016 | United Kingdom | Universal | 6,108 | 4.3 | 0.40% | 1.69% | 0.26%-0.65% |
| Seroprevalence and demographic factors associated with hepatitis B, hepatitis C and HIV infection from a hospital emergency department testing programme, London, United Kingdom, 2015 to 2016 | Bundle, et al; 2019 | 2015-2016 | United Kingdom | Universal | 6,211 | 2.0 | 1.2% (0.9%-1.8%) | 2.4% | 1.6% |
| Seroprevalence of HCV, HBV, and HIV in two inner-city London emergency departments | Cieply, et al; 2019 | 2015 | United Kingdom | Universal | 2,833 | 1.7 | 1.2% (0.9%-1.8%) | 2.0% | 1.3% |
| Detecting hepatitis infection in the emergency department | Hunter, et al; 2016 | 2016 | United Kingdom | Universal | 2,982 | 1.0 | 1.2% (0.9%-1.8%) | 1.2% |  |
| Incorporating HIV/hepatitis B virus/hepatitis C virus combined testing into routine blood tests in nine UK Emergency Departments: the “Going Viral” campaign | Orkin, et al; 2016 | 2014 | United Kingdom | Universal | 2,118 | 5.0 | 1.2% (0.9%-1.8%) |  | 1.8% |
| Vira+ Emic: opt-out hepatitis B and C testing in patients attending an urban emergency department | Wong, et al; 2019 |  | United Kingdom | Universal | 28,939 | 1.6 | 1.2% (0.9%-1.8%) | 1.9% | 1.1% |
| High prevalence of injection drug use and blood-borne viral infections among patients in an urban emergency department | Anderson, et al; 2020 | 2018-2019 | United States | Universal | 2,200 | 5.2 | 1.7% (1.4%-2.0%) | 8.8% |  |
| Implementation of a Collaborative HIV and Hepatitis C Screening Program in Appalachian Urgent Care Settings | Burrel, et al; 2018 | 2017-2018 | United States | Universal | 6,509 | 0.9 | 1.7% (1.4%-2.0%) | 1.6% | 0.2% |
| HCV screening, linkage to care, and treatment patterns at different sites across one academic medical center | Calner, et al; 2019 | 2016-2018 | United States | Universal | 13,829 | 7.7 | 1.7 (1.4%-2.0%) | 13.1% | 7.2% |
| Integrating HIV and Hepatitis C Screening in a High-Risk Emergency Department Population | Chechi, et al; 2019 | 2018-2019 | United States | Universal | 6,627 | 5.6 | 1.7 (1.4%-2.0%) | 9.6% | 3.8% |
| High Prevalence of Hepatitis C Infection Among Adult Patients at Four Urban Emergency Departments - Birmingham, Oakland, Baltimore, and Boston, 2015-2017 | Galbraith, et al; 2020 | 2015-2016 | United States | Universal | 14,252 | 5.4 | 1.7% (1.4%-2.0%) | 9.2% | 5.7% |
| Evaluation of the centers for disease control and prevention Recommendations for Hepatitis C virus testing in an urban emergency department | Hsieh, et al; 2016 | 2013 | United States | Universal | 4,713 | 13.8 | 0.8%-1.2% | 13.8% |  |
| Initial Outcomes of Universal HIV and HCV  Screening in a High Volume Academic Emergency Department | Hsu, et al; 2020 | 2020 | United States | Universal | 895 | 5.5 | 1.7% (1.4%-2.0%) | 9.3% | 3.8% |
| Prevalence of diagnosed and undiagnosed Hepatitis C in a midwestern urban emergency department | Lyons, et al; 2016 | 2008-2009 | United States | Universal | 924 | 8.2 | 1.7% (1.4%-2.0%) | 14% | 11.1% |
| HIV and HCV screening among trauma patients | Simoncini, et al; 2019 | 2016-2017 | United States | Universal | 1,160 | 8.2 | 1.7% (1.4%-2.0%) | 14% | 5.8% |
| Undiagnosed HIV and HCV Infection in a New York City Emergency Department, 2015 | Torian, et al; 2018 | 2015 | United States | Universal | 4,989 | 4.4 | 1.7% (1.4%-2.0%) | 7.5% (6.7%-8.2%) | 3.9% (2.8%-5.1%) |

*ED HVC prevalence over prevalence estimate of reference in the country-specific general population
