# Supplementary material for: Early cost-utility analysis of hepatitis C virus testing for emergency department attendees in France
Source: PLOS Glob Public Health. 2023 Feb 23;3(2):e0001559. doi: 10.1371/journal.pgph.0001559 (PMC10021824; doi:10.1371/journal.pgph.0001559)
Supplement: S1 Fig — (DOCX) [file pgph.0001559.s002.docx]

### S2 Fig Two-way sensitivity analysis


Proba.=probability. Note: bold values indicate base-case values: € 13 for anti-body test cost, 6% for background testing probability, 33% for proportion of individuals engaged in treatment.
