# Supplementary figures and images for: Early cost-utility analysis of hepatitis C virus testing for emergency department attendees in France
Source: PLOS Glob Public Health. 2023 Feb 23;3(2):e0001559. doi: 10.1371/journal.pgph.0001559 (PMC10021824; doi:10.1371/journal.pgph.0001559)

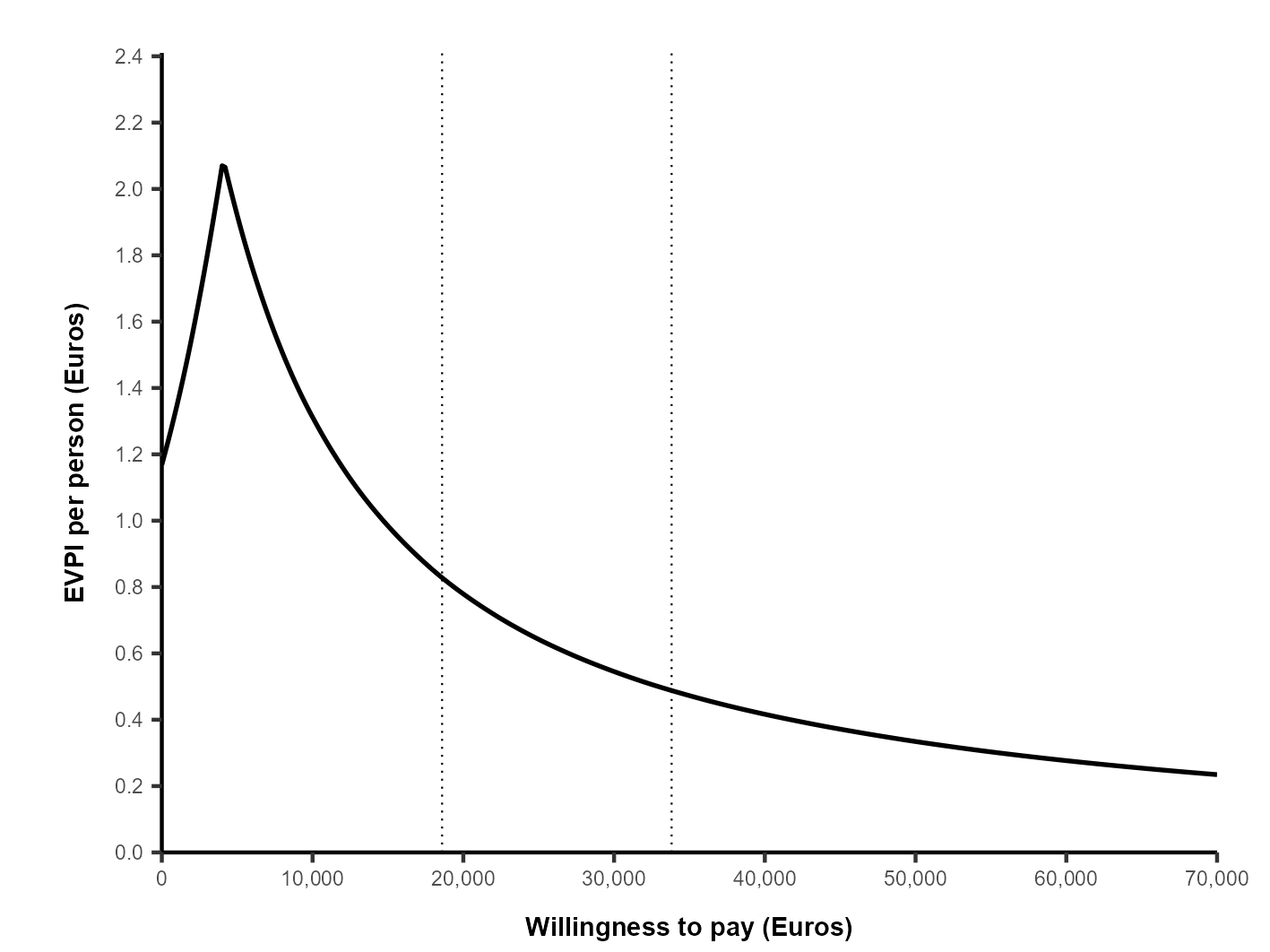

Supplement: S2 Fig — (TIFF) [file pgph.0001559.s003.tiff]
