# Supplementary material for: Early cost-utility analysis of hepatitis C virus testing for emergency department attendees in France
Source: PLOS Glob Public Health. 2023 Feb 23;3(2):e0001559. doi: 10.1371/journal.pgph.0001559 (PMC10021824; doi:10.1371/journal.pgph.0001559)
Supplement: S1 Table — (DOCX) [file pgph.0001559.s004.docx]

### S1 Table Population EVPI across a range of intervention time horizons, according to both willingness to pay thresholds

| **Time horizons** | **Willingness-to-pay** | |
| --- | --- | --- |
|  | **€ 18,592** | **€ 33,817** |
| **2 years** | € 17,441,366 | € 10,278,534 |
| **3 years** | € 25,844,313 | € 15,230,553 |
| **5 years** | € 42,040,355 | € 24,775,194 |
| **10 years** | € 79,197,903 | € 46,672,855 |

Estimated effective population assumed to be 10,662,000 different people attending the ED each year.
